# Chronoeffects of the Herbal Medicines *Puerariae radix* and *Coptidis rhizoma* in Mice: A Potential Role of REV-ERBα

**DOI:** 10.3389/fphar.2021.707844

**Published:** 2021-07-28

**Authors:** Jinming Liu, Haiman Xu, Li Zhang, Shuai Wang, Danyi Lu, Min Chen, Baojian Wu

**Affiliations:** ^1^ Department of Critical Care Medicine, Zhongshan Torch Development Zone Hospital, Zhongshan, China; ^2^ College of Pharmacy, Jinan University, Guangzhou, China; ^3^ Institute of Molecular Rhythm and Metabolism, Guangzhou University of Chinese Medicine, Guangzhou, China

**Keywords:** chronoefficacy, dosing time, puerariae radix, coptidis rhizoma, hyperhomocysteinemia, chronic colitis

## Abstract

Identifying drugs with dosing time-dependent effects (chronoeffects) and understanding the underlying mechanisms would help to improve drug treatment outcome. Here, we aimed to determine chronoeffects of the herbal medicines Puerariae radix (PR) and Coptidis rhizoma (CR), and investigate a potential role of REV-ERBα as a drug target in generating chronoeffects. The pharmacological effect of PR on hyperhomocysteinemia in mice was evaluated by measuring total homocysteine, triglyceride levels and lipid accumulation. PR dosed at ZT10 generated a stronger effect on hyperhomocysteinemia than drug dosed at ZT2. Furthermore, PR increased the expression levels of REV-ERBα target genes *Bhmt, Cbs* and *Cth* (encoding three key enzymes responsible for homocysteine catabolism), thereby alleviating hyperhomocysteinemia in mice. Moreover, CR attenuated chronic colitis in mice in a dosing time-dependent manner based on measurements of disease activity index, colon length, malondialdehyde/myeloperoxidase activities and IL-1β/IL-6 levels. ZT10 dosing generated a stronger anti-colitis effect as compared to ZT2 dosing. This was accompanied by lower production of colonic inflammatory cytokines (i.e., *Nlrp3*, *IL-1β*, *IL-6*, *Tnf-α* and *Ccl2*, REV-ERBα target genes) in colitis mice dosed at ZT10. The diurnal patterns of PR and CR effects were respectively consistent with those of puerarin (a main active constituent of PR, a REV-ERBα antagonist) and berberine (a main active constituent of CR, a REV-ERBα agonist). In addition, loss of *Rev-erbα* in mice abolished the dosing time-dependency in PR and CR effects. In conclusion, the therapeutic effects of PR and CR depend on dosing time in mice, which are probably attributed to diurnal expression of REV-ERBα as the drug target. Our findings have implications for improving therapeutic outcomes of herbal medicines with a chronotherapeutic approach.

## Introduction

Homocysteine (Hcy) is a sulfur-containing amino acid derived from methionine metabolism. It exists in both free and protein-bound forms, and can be oxidized in plasma to the disulfides Hcy-Hcy and Hcy-cysteine (Cattaneo, 1999). Free and protein-bound Hcy and its disulfides are globally referred to as total homocysteine (tHcy) (Cattaneo, 1999). Intracellular metabolism of Hcy occurs through two major pathways, namely, remethylation and transsulfuration. Deficiency of the metabolic enzymes [e.g., betaine homocysteine methyltransferase (BHMT), cystathionine β-synthase (CBS), and cystathionine *γ*-lyase (CTH)] involved in these two pathways can lead to hyperhomocysteinemia (Zhang et al., 2019). Elevated Hcy is considered to be an independent risk factor for cardiovascular and cerebrovascular diseases such as stroke and dementia, and therefore represents a major health problem (Mayer et al., 1996; Seshadri et al., 2002). Puerariae radix (PR, named “Gegen” in Chinese), the root of Pueraria lobata (Wild.) Ohwi, is a commonly used botanical drug in oriental medicine. It is used clinically to treat various cardiovascular diseases such as hypertension and angina pectoris and type 2 diabetes mellitus (Zhang et al., 2013). In our recent study, puerarin, a major active ingredient of PR, alleviates hyperhomocysteinemia in a dosing time-dependent manner (Chen et al., 2020). However, it remains to be determined whether PR can be used to combat hyperhomocysteinemia.

Inflammatory bowel diseases (IBD), including ulcerative colitis and Crohn’s disease, are chronic relapsing idiopathic diseases characterized by epithelial barrier damage and disruption of inflammatory homeostasis in the intestinal tract (Gupta et al., 2007; Wang et al., 2017). Abdominal pain, diarrhea, rectal bleeding and weight loss are the common symptoms in IBD patients (Cosnes et al., 2011; Sandborn et al., 2014). Although the pathogenic mechanisms for IBD remain largely unknown, it is generally believed that the disease is associated with dysregulated immune responses (De Souza and Fiocchi, 2016). A number of proinflammatory molecules [e.g., tumor necrosis factor-alpha (TNF-α), interleukin-1beta (IL-1β), and interleukin-6 (IL-6)] are expressed at high levels in the development of IBD (Hart et al., 2005; Brand et al., 2006). These cytokines have been shown to play an important role in mediating inflammatory responses. Currently, the main drugs for IBD management are anti-inflammatory agents, immune system suppressors and antibiotics. However, these drugs have some limitations such as poor treatment outcome and major adverse effects (Baumgart and Sandborn, 2007). Therefore, novel strategies are clearly needed to improve the treatment outcome for IBD.

Coptidis rhizoma (CR), also known as Huanglian in China, is the dried rhizome of medicinal plants from the family Ranunculaceae, including Coptis chinensis Franch (Weilian in Chinese), C. deltoidea C.Y. Cheng et Hsiao (Yalian in Chinese), and C. teeta Wall (Yunlian in Chinese) (Chen et al., 2008; Ma et al., 2012; Wang et al., 2019). It is a well-known herbal medicine and commonly used to treat diarrhoea, vomiting, abdominal fullness, high fever coma, jaundice, toothache, diabetes and eczema (Wu et al., 2015; Wang et al., 2019). To date, over 100 chemical constituents have been isolated and identified from CR. Alkaloids such as berberine are the most abundant ones among these chemical components and regarded as the main active ingredients of CR (Wang et al., 2019). In fact, berberine has been developed as a drug to treat gastrointestinal diseases including acute enteritis, diarrhea and colitis (Zhou and Mineshita, 2000; Tang et al., 2009; Lee et al., 2010). In a recent study, we have revealed diurnal pharmacological effects of berberine on chronic colitis in mice (Zhou et al., 2020). However, it remains unknown whether CR effects on colitis are dosing time-dependent or not.

Physiology and behaviors of organisms are subjected to circadian (∼24 h) rhythms due to daily changes in the environment (e.g., sunlight and temperature) (Foster and Roenneberg, 2008). Studies over the last two decades have identified the components of endogenous circadian clock systems that govern ∼24 h rhythms (persistent even in the absence of external cues such as light) (Reppert and Weaver, 2002). The circadian clock systems, consisting of several negative feedback loops, are present in hypothalamic suprachiasmatic nuclei (SCN) as well as in most peripheral tissues (e.g., liver and intestine). The core feedback loop comprises the core clock genes *BMAL1* (brain and muscle Arnt-like protein-1) and *CLOCK* (circadian locomotor output cycles kaput), whose protein products form a heterodimer and transcriptionally regulate rhythmic expressions of clock-controlled genes (CCGs) including *PER* (period), *CRY* (cryptochrome), and *REV-ERBα* (Chen et al., 2020). The SCN central clock, entrained by time cues (e.g., light), synchronizes the clocks in peripheral tissues via neural and endocrine signals (Dibner et al., 2010). REV-ERBα is a component of circadian clockwork and a ligand-responsive nuclear receptor. The synthetic compounds SR9009, SR8278, and GSK4112 as well as the natural compounds puerarin and berberine have been identified as REV-ERBα ligands (Chen et al., 2020; Zhou et al., 2020).

Chronotherapy, an emerging concept in the field of therapeutics, refers to a treatment strategy that aims to improve the overall control of a disease and to minimize side effects by altering the timing of medication (Kaur et al., 2013). It thus provides an approach to improve pharmacotherapy by administering drugs at the time-of-day when they are best effective and/or least toxic. Circadian variations in drug targets, severity of disease and pharmacokinetics are thought to be important factors affecting the efficacy of drugs (Chen et al., 2020; Zhou et al., 2020). For instance, temporal expression of the drug target REV-ERBα accounts for chronoeffects of the drugs such as puerarin and berberine (Chen et al., 2020; Zhou et al., 2020). In the present study, we aimed to determine circadian pharmacological effects of PR and CR, two common herbal medicines. PR and CR effects were assessed based on the diseases hyperhomocysteinemia and chronic colitis, respectively. We for the first time unraveled dosing time-dependent effects of PR and CR, which are potentially attributed to diurnal expression of the drug target REV-ERBα. It is proposed that therapeutic outcomes of PR and CR may be improved via timed delivery.

## Materials and Methods

### Materials

*Puerariae radix* (PR) and *Coptidis rhizoma* (CR) were obtained from the First Affiliated Hospital of Jinan University (Guangzhou, China) and were validated by the corresponding author. A voucher specimen (No. 201010-1) for PR and a voucher specimen (No. 201010-2) for CR were deposited at Research Center for Biopharmaceutics and Pharmacokinetics, College of Pharmacy, Jinan University. Puerarin (>98% purity, 3,681-99-0) and l-methionine (also referred as methionine in this paper, M101130) were purchased from Aladdin Chemicals (Shanghai, China). Berberine salt (also referred as berberine in this paper, >98% purity, 141,433-60-5) was purchased from TopScience Co. (Shanghai, China). Dextran sulfate sodium (DSS, molecular weight of 36–50 kDa, MP0216011080) was purchased from MP Biomedicals (Irvine, CA). Assay kits for total homocysteine (tHcy), triglyceride (TG), malondialdehyde (MDA), and myeloperoxidase (MPO) were purchased from Jiancheng Bioengineering Institute (Nanjing, China). Enzyme-linked immunosorbent assay (ELISA) kits for IL-1β and IL-6 were purchased from Neobioscience Technology (Shenzhen, China).

### Animals

Wild-type C57BL/6 mice (male, 5–6 weeks of age) were purchased from HFK Biotechnology (Beijing, China). *Rev-erbα* ^*−/−*^ mice on a C57BL/6 background have been established and validated in our laboratory (Zhang et al., 2019). Mice were maintained under a 12 h light/12 h dark cycle (lights on at 7:00 AM (=Zeitgeber time 0/ZT0) and lights off at 7:00 PM (=ZT12)), with free access to food and water. All experiments were performed using protocols approved by the Institutional Animal Care and Use Committees of Guangzhou University of Chinese Medicine (Appr. date: 2020-09-09; IACUC Issue No: ZYD-2020-64). All surgery was performed under isoflurane anesthesia, and efforts were made to minimize suffering.

### Extraction of Herbal Medicines

**FIGURE 1:**
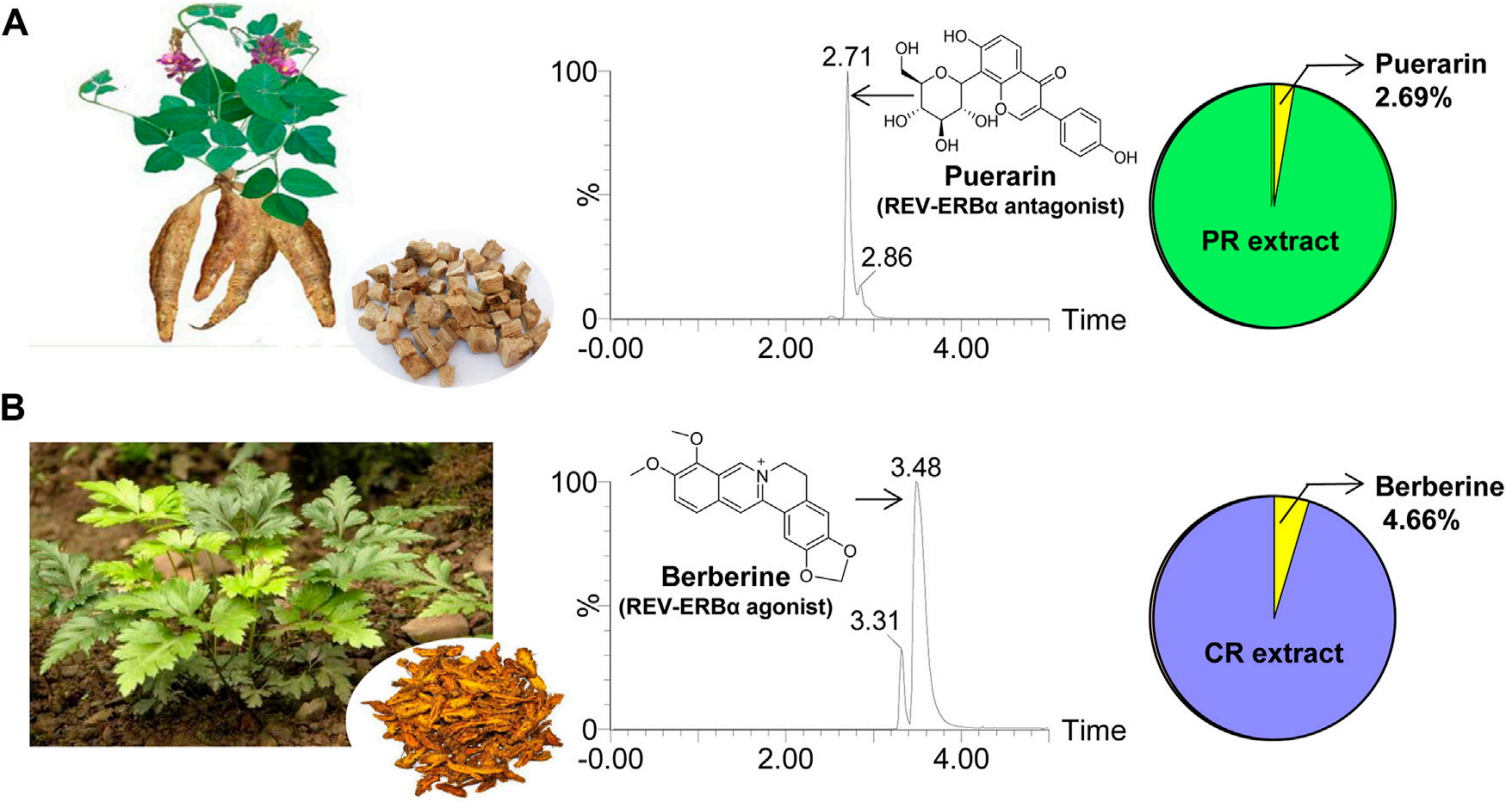
Characterization of PR and CR. **(A)** Whole plant and rhizome of PR **(left)**, representative extracted ion chromatogram of puerarin **(middle)** and the percentage of puerarin in PR extract derived from UPLC-QTOF/MS analysis **(right)**. **(B)** Whole plant and rhizome of CR **(left)**, representative extracted ion chromatogram of berberine **(middle)** and the percentage of berberine in CR extract derived from UPLC-QTOF/MS analysis **(right)**.

Extracts of PR and CR were prepared according to previously reported methods (Jeong et al., 2014; Chang et al., 2015). In brief, raw PR (20 g) was ground into fine powder, extracted two times (1 h each time) with 30% ethanol (150 ml) under boiled water bath, and filtered by filter paper (Chang et al., 2015). The filtrate was dried in vacuum at 50°C. The fine powder of CR (20 g) was extracted two times (1 h each time) with 80% ethanol (150 ml) under boiled water bath, and filtered by filter paper (Jeong et al., 2014). The filtrate was dried in vacuum at 50°C. The extraction yields were 6.25 and 12.5% for PR and CR, respectively. Puerarin accounts for 2.69% (w/w) of total PR exact, and berberine accounts for 4.66% (w/w) of total CR exact (Figure 1).

### Sample Preparation for Qualitative Analysis

Approximately 0.4 g of PR and CR extracts were dissolved in 10 ml 50% methanol (*v/v*), followed by vortex and centrifugation (13,000 g, 15 min). The supernatant was filtered through 0.22 μm membranes, and then diluted to appropriate concentration (i.e., 2 mg/ml) for analysis.

### UPLC-QTOF/MS Analysis

Puerarin in PR and berberine in CR were quantified as previously described (Chen et al., 2020; Zhou et al., 2020). Peak areas of puerarin and berberine were recorded with extract masses of *m/z* 417.05 ± 0.05 Da and 336.06 ± 0.05 Da, respectively. Representative extracted ion chromatograms are provided in Figure 1.

### Methionine-Induced Hyperhomocysteinemia and Drug Treatment

Hyperhomocysteinemia was induced by feeding mice with 0.5% methionine in drinking water for 8 weeks as previously described (Chen et al., 2020). Normal control mice were provided with pure drinking water. Wild-type mice with hyperhomocysteinemia were divided into eight groups randomly (*n =* 5 per group) to assess the effects of PR on hyperhomocysteinemia. For dose-response study, three groups of mice were respectively gavaged with 62.5, 125 and 188 mg/kg PR extract (equivalent to 1, 2, and 3 g/kg raw herb, respectively) at ZT10 once a day for 7 days. Control group of mice were treated with vehicle. On day 8, mice were sacrificed at ZT2 to collect plasma and liver samples. For chronoeffect study, the remaining groups of mice were gavaged with 125 mg/kg PR extract or vehicle at ZT2 or ZT10 per day for 7 days. On day 8, mice were sacrificed at ZT2 to collect plasma, liver and colon samples. *Rev-erbα* ^*−/−*^ mice with hyperhomocysteinemia were randomly divided into four groups (*n =* 5 per group) to assess the role of REV-ERBα in PR chronoeffect. These mice were gavaged with 125 mg/kg PR extract or vehicle at ZT2 or ZT10 per day for 7 days. On day 8, mice were sacrificed at ZT2 to collect plasma and liver samples.

### DSS-Induced Colitis and Drug Treatment

Chronic colitis was induced in mice via three cycles of 2% DSS (in drinking water) feeding as previously described (Zhou et al., 2020). Control mice were fed with pure drinking water. The colitis mice (wild-type) were divided into eight groups (namely, groups 1–8, *n* = 6 per group). Group 1, group 2, and group 3 of mice were respectively gavaged with 12.5, 25, and 50 mg/kg CR extract (equivalent to 100, 200, and 400 mg/kg raw herb, respectively) at ZT10 once a day for 7 days. Group 4 of mice were treated with vehicle. On day 8, CR-, vehicle-treated and normal control mice were sacrificed at ZT2 to collect plasma and colon samples. The remaining groups of mice were gavaged with 50 mg/kg CR extract or vehicle at ZT2 or ZT10 once a day for 7 days. On day 8, mice were sacrificed at ZT2 to collect plasma, colon and liver samples. Water intake was measured to the nearest ml with calibrated Richter tubes and recorded daily at ZT0 and ZT12 during CR treatment. Food intake was determined by providing preweighed food in excess to the feeding cage and weighing leftover food daily at ZT0 and ZT12 during CR treatment. *Rev-erbα* ^*−/−*^ mice with colitis were randomly divided into four groups (*n =* 6 per group) to assess the role of REV-ERBα in CR chronoeffect. These mice were gavaged with 50 mg/kg CR extract or vehicle at ZT2 or ZT10 per day for 7 days. On day 8, mice were sacrificed at ZT2 to collect plasma and colon samples.

### Biochemical Analysis

Plasma and hepatic tHcy levels were quantified by performing enzymatic cycling assays with a homocysteine kit according to the manufacturer’s protocol (Jiancheng Bioengineering Institute, Nanjing, China). Plasma and hepatic TG levels were measured using a TG assay kit according to the manufacturer’s protocol (Jiancheng Bioengineering Institute, Nanjing, China). Colonic MDA and MPO activities were measured using malondialdehyde and myeloperoxidase assay kits according to the manufacturer’s protocol (Jiancheng Bioengineering Institute, Nanjing, China). Colonic IL-1β and IL-6 levels were quantified with ELISA kits according to the manufacturer’s protocol (Neobioscience, Shenzhen, China).

### Oil Red O Staining

Oil red O staining was performed to analyze hepatic lipid accumulation as previously described (Chen et al., 2020). In brief, liver samples were fixed in 10% paraformaldehyde and embedded in paraffin. Paraffin sections (10 μm) were sequentially stained with oil red O and hematoxylin. Images were obtained using a Zeiss Axio Imager M1 microscope (Carl Zeiss AG, Oberkochen, Germany).

### Macroscopic Scoring of Colitis

Changes in body weight, diarrhea and bleeding in colitis mice were recorded on a daily basis after drug treatment. Scoring was performed according to the criteria described previously (Sann et al., 2013). Body weight changes were calculated relative to day 1. Disease activity index (DAI) is the sum of the weight loss, diarrhea and bloody stool scores. Colons lengths (as an indirect marker of colonic inflammation) were measured with a centimeter ruler.

### Hematoxylin-Eosin Staining

Colon tissues were fixed in 4% paraformaldehyde and embedded in paraffin, followed by hematoxylin-eosin (H&E) staining. Histological damage was scored based on enterocyte loss, crypt inflammation, *lamina propria* mononuclear cells, neutrophil infiltration, and epithelial hyperplasia as previously described (Song et al., 2016). The total histological score ranged from 0 (no changes) to 6 (extensive cell infiltration and tissue damage).

### qPCR

qPCR assays were performed as described in our previous publication (Guo et al., 2018). Amplification reaction consisted of an initial denaturation at 95°C for 5 min, followed by 40 cycles of denaturation at 95°C for 10 s, annealing at 60°C for 30 s, and extension at 72°C for 30 s. Primers are listed in Table 1.

**TABLE 1:** Primers used in this study.

	Forward (5′-3′ sequence)	Reverse (5′-3′ sequence)
*Bhmt*	TTA​GAA​CGC​TTA​AAT​GCC​GGA​G	GAT​GAA​GCT​GAC​GAA​CTG​CCT
*Cbs*	GGG​ACA​AGG​ATC​GAG​TCT​GGA	AGC​ACT​GTG​TGA​TAA​TGT​GGG
*Cth*	TTC​CTG​CCT​AGT​TTC​CAG​CAT	GGA​AGT​CCT​GCT​TAA​ATG​TGG​TG
*Ppib*	TCC​ACA​CCC​TTT​TCC​GGT​CC	CAA​AAG​GAA​GAC​GAC​GGA​GC
*Ccl2*	CCA​CAA​CCA​CCT​CAA​GCA​CT	AGG​CAT​CAC​AGT​CCG​AGT​CA
*Tnf-α*	AGG​GTC​TGG​GCC​ATA​GAA​CT	CCA​CCA​CGC​TCT​TCT​GTC​TAC
*Nlrp3*	ATT​ACC​CGC​CCG​AGA​AAG​G	TCG​CAG​CAA​AGA​TCC​ACA​CAG
*IL-1β*	AAT​GCC​ACC​TTT​TGA​CAG​TGA​TG	AGC​TTC​TCC​ACA​GCC​ACA​AT
*IL-6*	ATC​CAG​TTG​CCT​TCT​TGG​GAC​TGA	TAA​GCC​TCC​GAC​TTG​TGA​AGT​GGT
*Hmbs*	AAG​GGC​TTT​TCT​GAG​GCA​CC	AGT​TGC​CCA​TCT​TTC​ATC​ACT​G

### Statistical Analysis

Data are presented as mean ± SD (standard deviation). Student’s t test was used to test for statistical differences between two groups. One-way or two-way ANOVA followed by Bonferroni post hoc test was used for multiple group comparisons. The level of significance was set at *p* < 0.05 (*).

## Results

### PR Dose-dependently Alleviates Hyperhomocysteinemia in Mice

**FIGURE 2:**
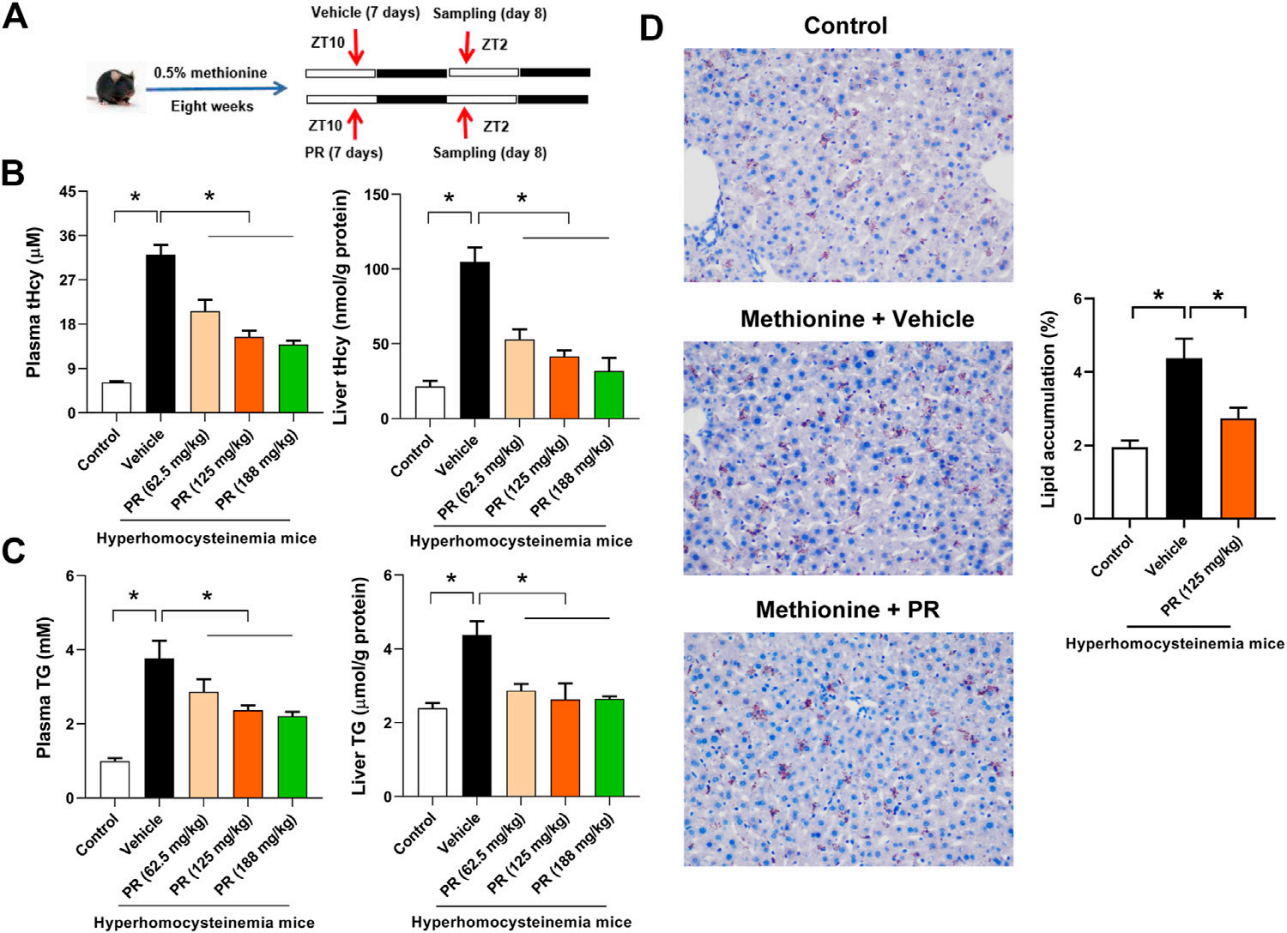
PR dose-dependently alleviates hyperhomocysteinemia in mice. **(A)** Schematic diagram for the experimental protocol of hyperhomocysteinemia model establishment and PR treatment. **(B)** Plasma and hepatic tHcy levels in each group of mice. Data are mean ± SD (*n* = 5). *p < 0.05 (one-way ANOVA with Bonferroni post hoc test). **(C)** Plasma and hepatic TG levels in each group of mice. Data are mean ± SD (*n* = 5). *p < 0.05 (one-way ANOVA with Bonferroni post hoc test). **(D)** Oil red O staining of livers from control and hyperhomocysteinemia mice. Data are mean ± SD (*n* = 5). *p < 0.05 (*t*-test).

Hyperhomocysteinemia in mice was induced by feeding 0.5% methionine in drinking water for 8 weeks as described in our prior study (Chen et al., 2020) (Figure 2A)*.* Mice with hyperhomocysteinemia showed increased circulating and hepatic levels of both tHcy and TG, suggesting successful construction of hyperhomocysteinemia model (Figure 2B,C). Elevated lipid accumulation in the liver was confirmed by oil red O staining (Figure 2D). Puerarin, a major constituent of PR, was shown to possess an anti-hyperhomocysteinemia effect in our prior study (Chen et al., 2020). We thus investigated whether PR alleviates hyperhomocysteinemia. The daily therapeutic dose of PR (raw herb) is 10–15 g for adults (Chinese Pharmacopoeia, 2015). According to dose translation between humans and mice, the equivalent dose for mice was estimated to be 2.05–3.075 g/kg (raw herb) per day (Nair et al., 2018). Therefore, three different doses (i.e., 62.5, 125, and 188 mg/kg PR extract, equivalent to 1, 2, and 3 g/kg raw herb, respectively) were selected to test the effects of PR on hyperhomocysteinemia. Hyperhomocysteinemia mice were treated with RP extract or vehicle by oral gavage at ZT10 once a day for 7 days (Figure 2A). PR significantly decreased the levels of plasma and hepatic tHcy in a dose-dependent manner (Figure 2B). In the meantime, plasma and hepatic levels of TG were reduced by PR (Figure 2C). Reduction of hepatic lipid accumulation by PR extract (125 mg/kg) in hyperhomocysteinemia mice was also illustrated by oil red O staining (Figure 2D). Altogether, these data indicate alleviation of hyperhomocysteinemia by PR in mice.

### Chronoeffect of PR on Hyperhomocysteinemia in Mice

**FIGURE 3:**
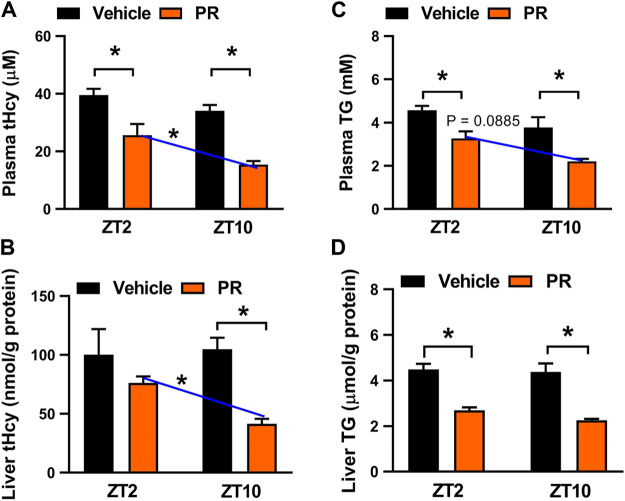
Chronoeffect of PR on hyperhomocysteinemia in mice. **(A)** Effects of PR (125 mg/kg) on plasma tHcy levels at two different dosing times ZT2 and ZT10. **(B)** Effects of PR (125 mg/kg) on hepatic tHcy levels at two different dosing times ZT2 and ZT10. **(C)** Comparisons of plasma TG levels for drug (125 mg/kg) treatments at ZT2 and ZT10. **(D)** Comparisons of hepatic TG levels for drug (125 mg/kg) treatments at ZT2 and ZT10. Data are mean ± SD (*n* = 5). *p < 0.05 (two-way ANOVA with Bonferroni post hoc test).

We next examined potential impact of dosing time on PR effects because the major constituent puerarin displays a dosing time-dependent effect (Chen et al., 2020). To this end, hyperhomocysteinemia mice were treated with PR extract (125 mg/kg, once daily for 7 days) at each of two time points ZT2 (corresponding to morning) and ZT10 (corresponding to evening). PR dosed at either ZT2 or ZT10 decreased the plasma and hepatic levels of both tHcy and TG (Figure 3). However, ZT10 dosing generated a superior effect as compared to ZT2 dosing, indicating chronoeffect of PR on hyperhomocysteinemia (Figure 3).

**FIGURE 4:**
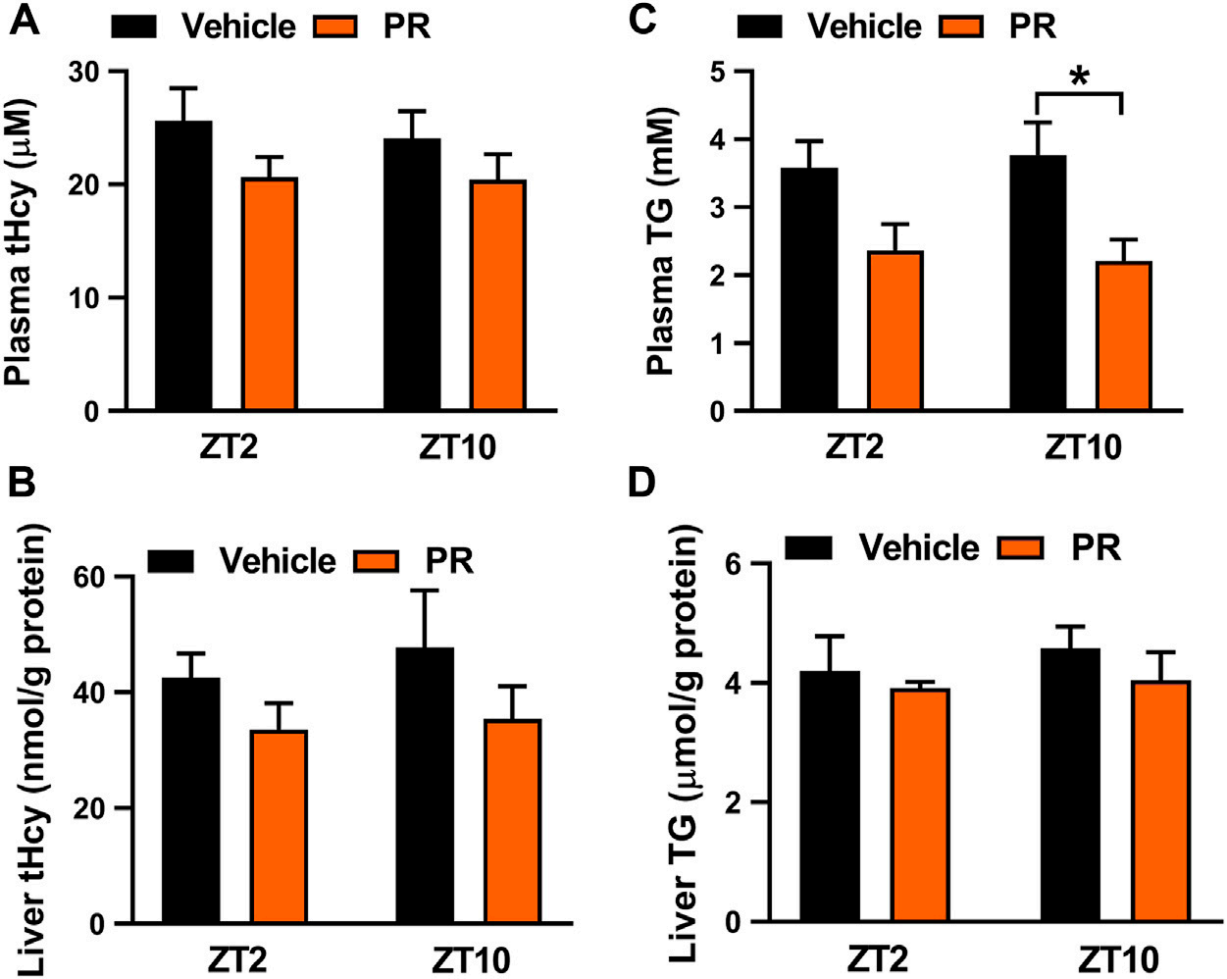
Rev-erbα ablation in mice abrogated the chronoeffect of PR on hyperhomocysteinemia. **(A)** Effects of PR (125 mg/kg) on plasma tHcy levels at two different dosing times ZT2 and ZT10 in *Rev-erbα* ^*−/−*^ mice with hyperhomocysteinemia. **(B)** Effects of PR (125 mg/kg) on hepatic tHcy levels at two different dosing times ZT2 and ZT10 in *Rev-erbα* ^*−/−*^ mice with hyperhomocysteinemia. **(C)** Comparisons of plasma TG levels for drug (125 mg/kg) treatments at ZT2 and ZT10 in *Rev-erbα* ^*−/−*^ mice with hyperhomocysteinemia. **(D)** Comparisons of hepatic TG levels for drug (125 mg/kg) treatments at ZT2 and ZT10 in *Rev-erbα* ^*−/−*^ mice with hyperhomocysteinemia. Data are mean ± SD (*n* = 5). *p < 0.05 (two-way ANOVA with Bonferroni post hoc test).

**FIGURE 5:**
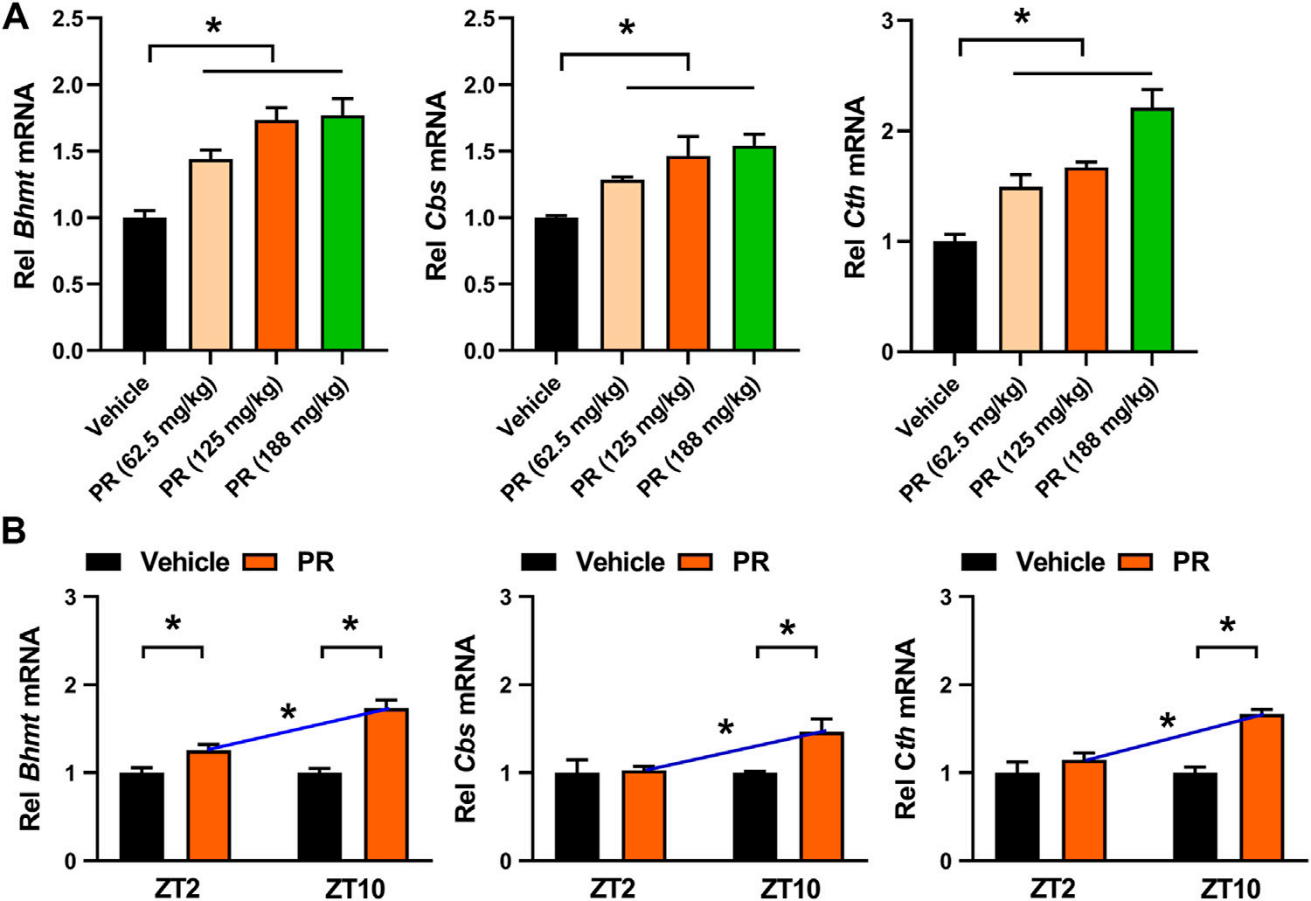
PR induces hepatic *Bhmt, Cbs* and *Cth* expressions in hyperhomocysteinemia mice. **(A)** Effects of PR (62.5, 125, and 188 mg/kg) on *Bhmt, Cbs,* and *Cth* mRNA expressions in hyperhomocysteinemia mice. *p < 0.05 (one-way ANOVA with Bonferroni post hoc test). Rel, Relative. **(B)** Effects of PR (125 mg/kg) on *Bhmt, Cbs,* and *Cth* mRNA expressions at ZT2 and ZT10. Data are mean ± SD (*n* = 5). *p < 0.05 (two-way ANOVA with Bonferroni post hoc test). Rel, Relative.

The time-varying effect of PR was in full agreement with that of puerarin, the main active compound of PR (Chen et al., 2020). Puerarin acts on the circadian protein REV-ERBα (whose expression oscillates with times of the day with a higher level at ZT10 and a lower level at ZT2) as an antagonist to increase hepatic expressions of three key enzymes involved in homocysteine catabolism (i.e., BHMT, CBS, and CTH), thereby alleviating hyperhomocysteinemia in mice (Chen et al., 2020; Zhang et al., 2019). Because the drug target is an oscillating protein, puerarin displays a chronoeffect (with a superior effect at ZT10 than at ZT2). To test whether the dosing time-dependent effects of PR in mice are associated with puerarin and REV-ERBα, we determined plasma and hepatic levels of tHcy and TG in *Rev-erbα* ^*−/−*^ mice with hyperhomocysteinemia after 7 days’ PR treatment. Intriguingly, PR treatment slightly decreased the levels of both tHcy and TG in *Rev-erbα* ^*−/−*^ mice (Figure 4). *Rev-erbα* ablation abrogated the time-dependency in PR effect (reflected by similar levels of tHcy and TG) (Figure 4). In addition, we determined the expression levels of hepatic *Bhmt, Cbs* and *Cth* in PR- or vehicle-treated hyperhomocysteinemia mice. PR treatment dose-dependently increased the levels of *Bhmt, Cbs* and *Cth* in hyperhomocysteinemia mice (Figure 5A). Moreover, ZT10 dosing generated higher levels of *Bhmt, Cbs* and *Cth* than at ZT2 dosing in hyperhomocysteinemia mice (Figure 5B). The higher *Bhmt, Cbs* and *Cth* expression for ZT10 dosing may contribute to the stronger drug effects. Diurnal rhythms in drug absorption and metabolism may result in time-dependent exposure, and thus in time-dependent drug effects (Zhang et al., 2018a; Yu et al., 2019). However, we observed no significant difference in systemic exposure or liver distribution of puerarin between ZT2 and ZT10 dosing of PR (Supplementary Figure S1). It was noted that PR treatment did not affect the expression of inflammatory factors (i.e., *Nlrp3, IL-1β, IL-6, Tnf-α* and *Ccl2*) in the colon (Supplementary Figure S4A)*.* Taken together, the pharmacological effect of PR on hyperhomocysteinemia is dosing time-dependent in mice. This is potentially attributed to diurnal expression of the drug target REV-ERBα.

### CR Dose-dependently Attenuates Chronic Colitis in Mice

**FIGURE 6:**
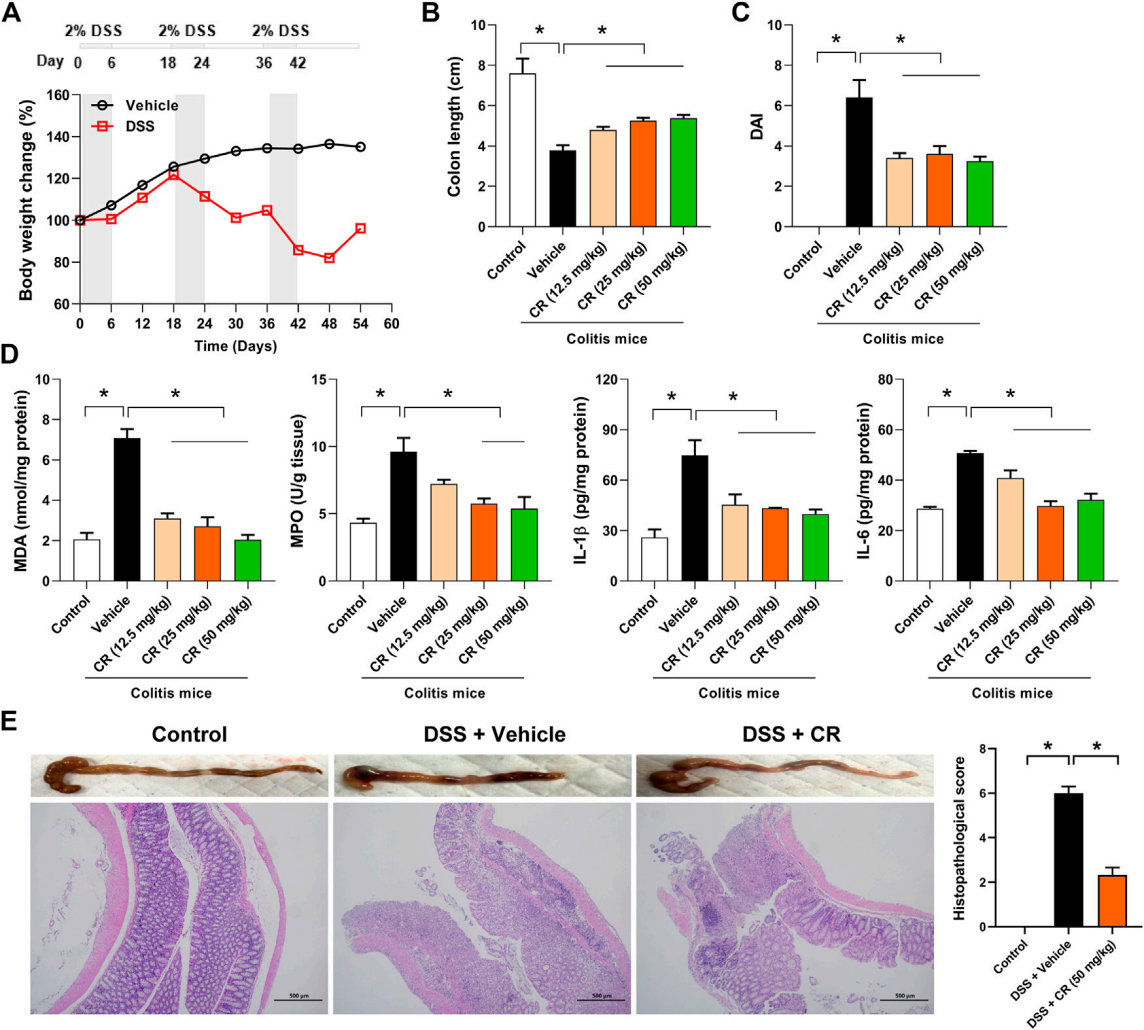
CR dose-dependently attenuates chronic colitis in mice. **(A)** Schematic diagram for the experimental protocol of chronic colitis establishment with mice. The bottom panel shows the mean weight change in control and DSS groups of mice. **(B)** Colons lengths of control mice and colitis mice treated CR. Data are mean ± SD (*n* = 6). *p < 0.05 (one-way ANOVA with Bonferroni post hoc test). **(C)** DAI scores of control mice and colitis mice treated CR. DAI was scored based on diarrhea, bleeding and body weight loss. Data are mean ± SD (*n* = 6). *p < 0.05 (one-way ANOVA with Bonferroni post hoc test). **(D)** Measurements of colonic MDA/MPO activities and IL-1β/IL-6 levels. Data are mean ± SD (*n* = 6). *p < 0.05 (one-way ANOVA with Bonferroni post hoc test). **(E)** Macroscopic appearance and H&E staining of colons as well as histopathological scores, showing an anti-colitis effect of CR (50 mg/kg). Data are mean ± SD (*n* = 6). *p < 0.05 (*t*-test).

To assess the therapeutic effect of CR on chronic colitis, we established mouse model with DSS-induced chronic colitis following a published protocol (Figure 6A) (Zhou et al., 2020). The mice showed significant loss of body weight after the second cycle of DSS treatment (Figure 6A). After three cycles of DSS administration, mice with colitis showed reduced colon length (a marker of intestinal inflammation) as well as increased levels of DAI, MDA (a measure of the colonic oxidative insult), MPO (an index of neutrophil accumulation), and inflammatory factors (IL-1β and IL-6) (Figure 6B,C,D). The colonic injury in colitis mice was also confirmed by H&E staining (Figure 6E). As expected, CR treatment dose-dependently increased the colon length in colitis mice (Figure 6B). Moreover, CR-treated colitis mice exhibited a decreased DAI score and gained body weight compared with vehicle-treated mice (Figure 6C and Supplementary Figure S3A). In addition, biochemical assessments showed that colonic MDA and MPO activities as well as colonic IL-1β and IL-6 levels were reduced by CR in a dose-dependent manner (Figure 6D). Alleviation of colitis by CR was further verified by histopathological analysis of the colon sections (Figure 6E). To be specific, enterocyte loss, crypt inflammation, *lamina propria* mononuclear cells, neutrophil infiltration, and epithelial hyperplasia were significantly lower in CR-treated than in vehicle-treated colitis mice (Figure 6E) (Song et al., 2016). Taken together, these findings indicate that CR can reduce the severity of chronic colitis in mice.

### Chronoeffect of CR on Chronic Colitis in Mice

**FIGURE 7:**
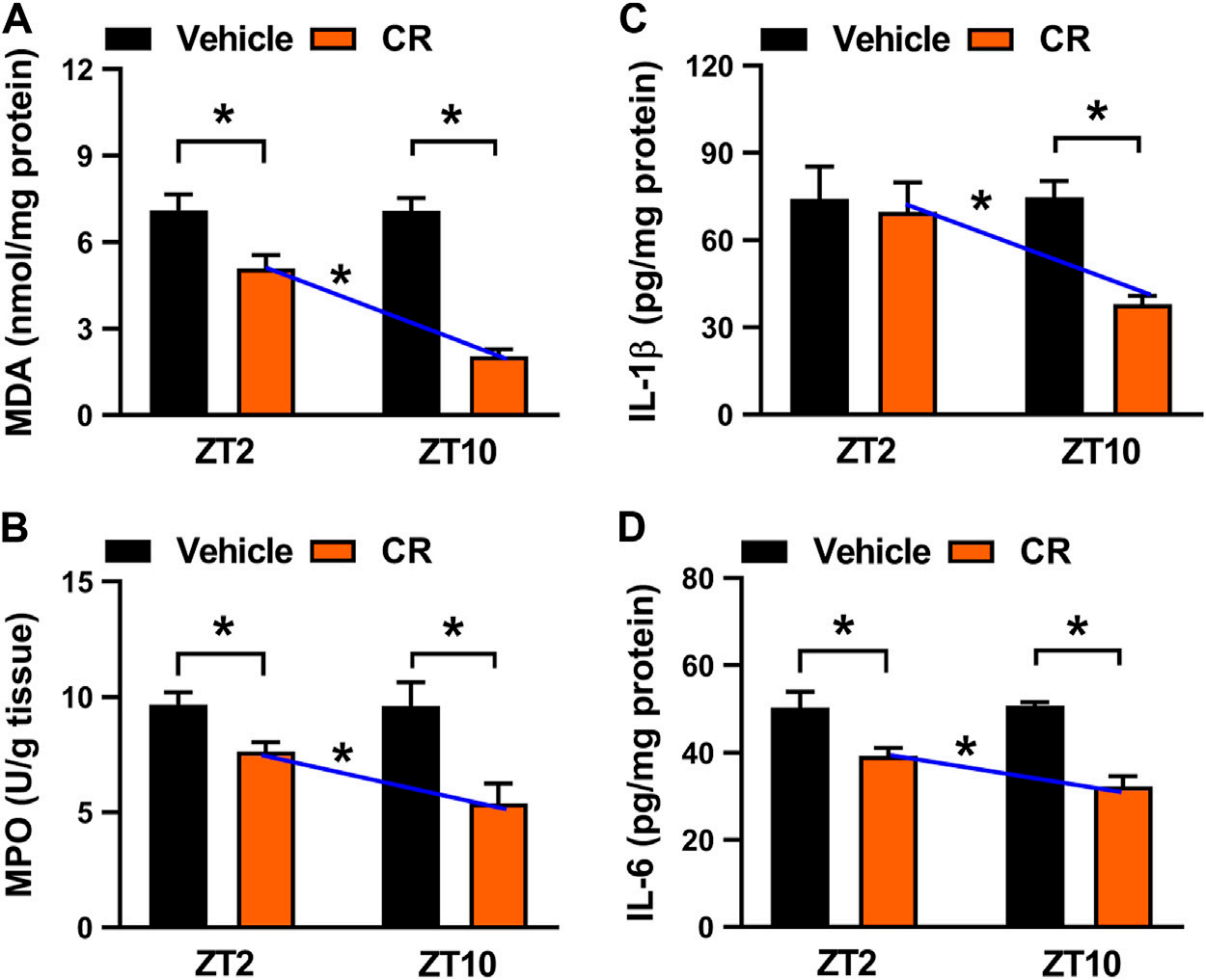
Circadian time-dependent effects of CR on chronic colitis in mice. **(A)** Effects of CR (50 mg/kg) on colonic MDA activities at ZT2 and ZT10. **(B)** Effects of CR (50 mg/kg) on colonic MPO activities at ZT2 and ZT10. **(C)** Comparisons of colonic IL-1β levels for drug (50 mg/kg) treatments at ZT2 and ZT10. **(D)** Comparisons of colonic IL-6 levels for drug (50 mg/kg) treatments at ZT2 and ZT10. Data are mean ± SD (*n* = 6). *p < 0.05 (two-way ANOVA with Bonferroni post hoc test).

We next examined potential impact of dosing time on CR effect because the major constituent berberine displays a dosing time-dependent effect (Zhou et al., 2020). To this end, colitis mice were gavaged with CR extract (50 mg/kg, once daily for 7 days) at ZT2 or ZT10. CR treatment did not affect daily food or water intake in mice (Supplementary Figures S3B,C). CR dosing at ZT2 and at ZT10 significantly decreased colonic MDA and MPO activities (Figure 7A,B). Additionally, CR dosed at either ZT2 or ZT10 reduced the levels of IL-1β and/or IL-6 (Figure 7C,D). Interestingly, we found that the anti-colitis effects of CR were stronger when the herbal medicine was dosed at ZT10, and were weaker when drug was dosed at ZT2 (Figure 7). Therefore, CR attenuates chronic colitis in a dosing time-dependent manner in mice with a stronger effect at ZT10.

**FIGURE 8:**
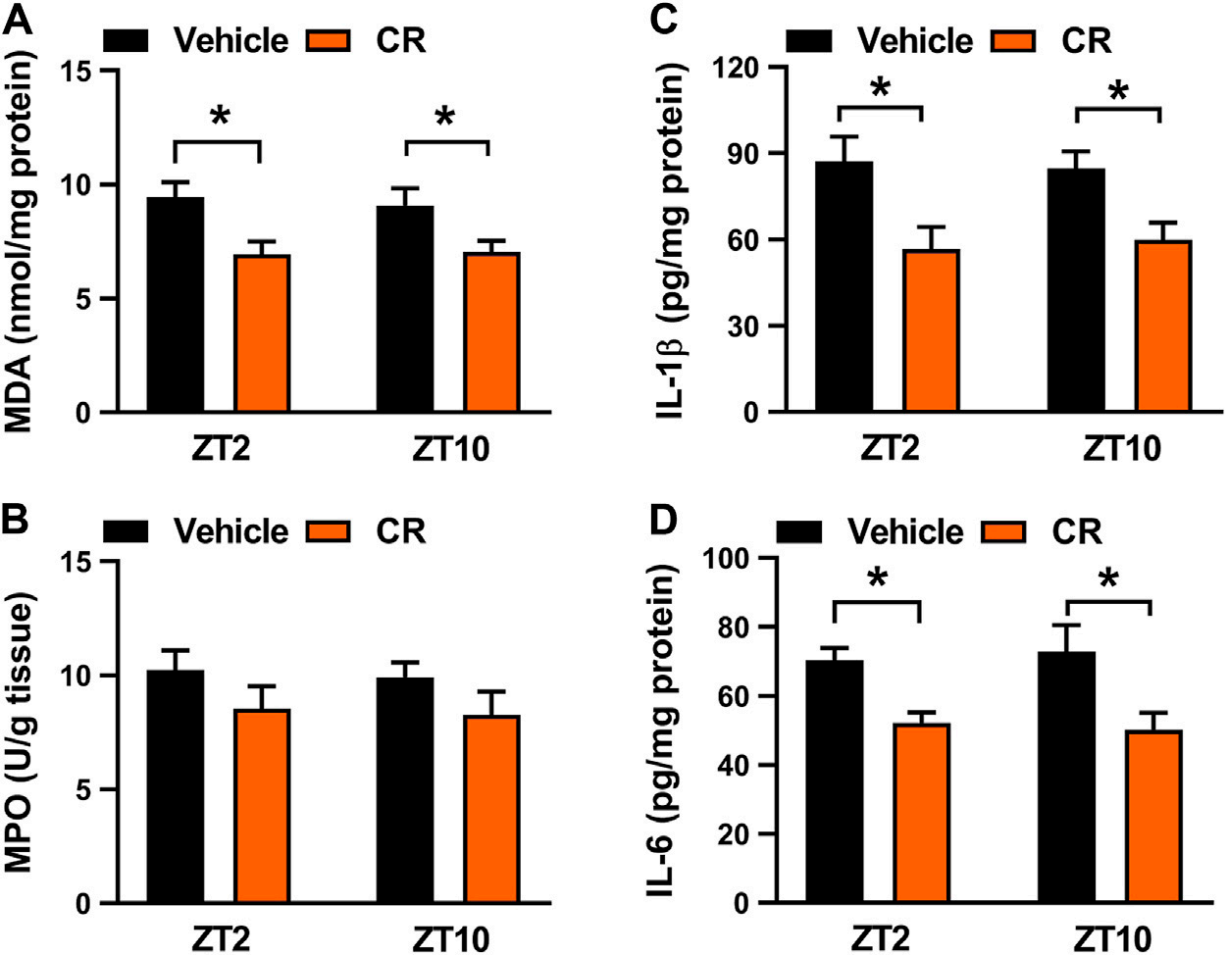
*Rev-erbα* ablation in mice abrogated dosing time-dependency of CR effect on colitis. **(A)** Effects of CR (50 mg/kg) on colonic MDA activities at ZT2 and ZT10 in *Rev-erbα* ^*−/−*^ mice with colitis. **(B)** Effects of CR (50 mg/kg) on colonic MPO activities at ZT2 and ZT10 in *Rev-erbα* ^*−/−*^ mice with colitis. **(C)** Comparisons of colonic IL-1β levels for drug (50 mg/kg) treatments at ZT2 and ZT10 in *Rev-erbα* ^*−/−*^ mice with colitis. **(D)** Comparisons of colonic IL-6 levels for drug (50 mg/kg) treatments at ZT2 and ZT10 in *Rev-erbα* ^*−/−*^ mice with colitis. Data are mean ± SD (*n* = 6). *p < 0.05 (two-way ANOVA with Bonferroni post hoc test).

**FIGURE 9:**
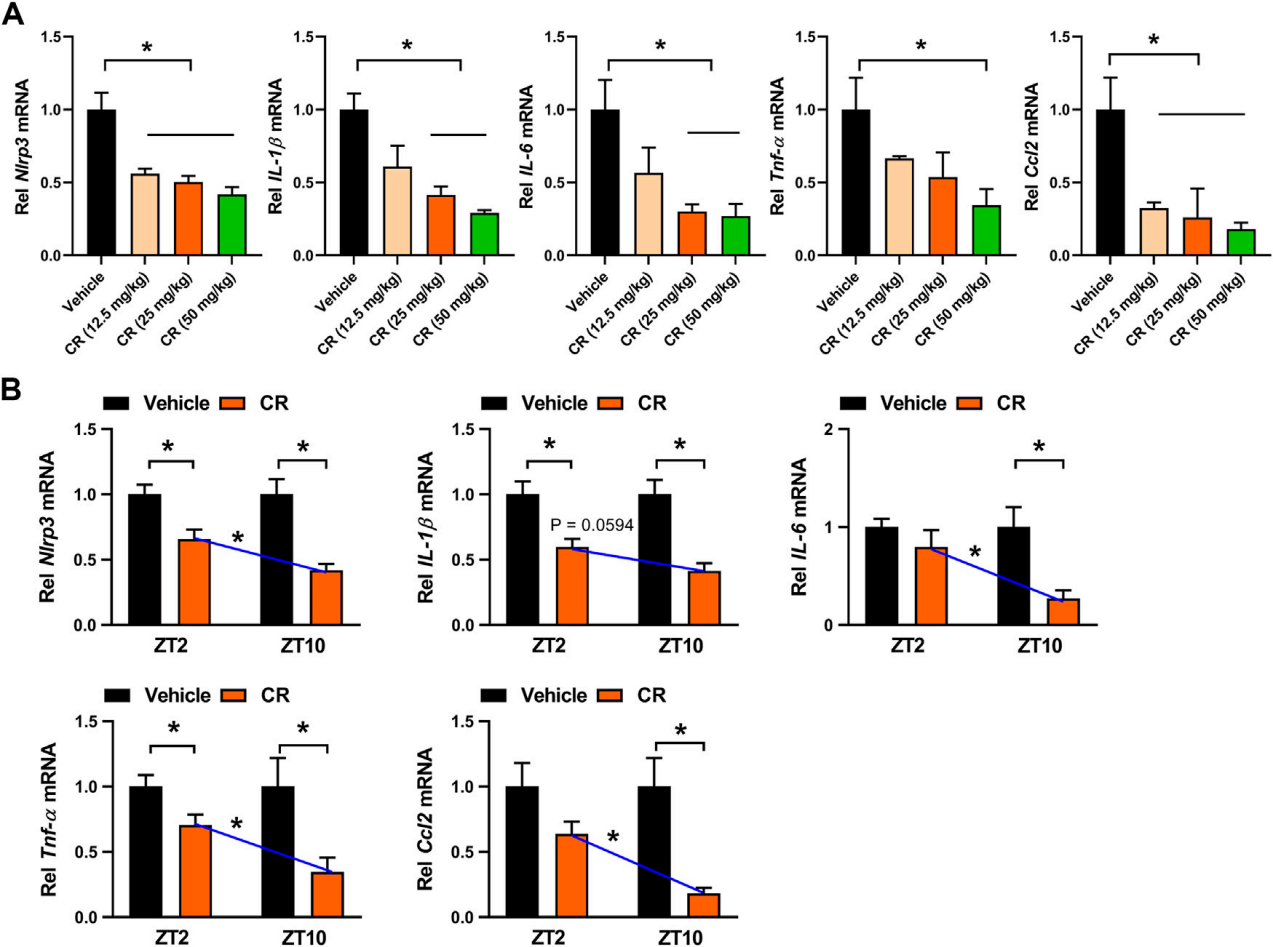
CR down-regulates colonic inflammatory cytokines in colitis mice. **(A)** Effects of CR (12.5, 25 and 50 mg/kg) on *Nlrp3, IL-1β, IL-6, Tnf-α* and *Ccl2* mRNA expressions in colitis mice. *p < 0.05 (one-way ANOVA with Bonferroni post hoc test). **(B)** Effects of CR (50 mg/kg) on *Nlrp3, IL-1β, IL-6, Tnf-α* and *Ccl2* mRNA expressions at ZT2 and ZT10. Data are mean ± SD (*n* = 6). *p < 0.05 (two-way ANOVA with Bonferroni post hoc test). Rel, Relative.

Berberine acts on the circadian protein REV-ERBα as an agonist to restrain colonic inflammation via intercepting NF-κB signaling and NLRP3 inflammasome activation (Wang et al., 2017; Zhou et al., 2020). Because the drug target is an oscillating protein, berberine displays a chronoeffect on chronic colitis (with a superior effect at ZT10 than at ZT2). To determine whether the dosing time-dependent anti-colitis effects of CR in mice are associated with berberine and REV-ERBα, we measured colonic MDA and MPO activities as well as IL-1β and IL-6 levels in *Rev-erbα* ^*−/−*^ mice with colitis after 7 days’ CR treatment. *Rev-erbα* ablation abrogated the dosing time-dependency in CR effect as evidenced by similar MDA and MPO activities as well as IL-1β and IL-6 levels between ZT2 and ZT10 dosing (Figure 8). Next, we examined the mRNA levels of REV-ERBα target genes (i.e., *Nlrp3, IL-1β, IL-6, Tnf-α,* and *Ccl2*) which are key inflammatory cytokines in colon tissue (Wang et al., 2017; Zhou et al., 2020)*.* CR dose-dependently down-regulated *Nlrp3, IL-1β, IL-6, Tnf-α* and *Ccl2* mRNA levels in colitis mice (Figure 9A). Moreover, CR (50 mg/kg) dosing at ZT2 and ZT10 caused decreases in *Nlrp3, IL-1β, IL-6, Tnf-α* and *Ccl2* mRNAs (Figure 9B). However, the changes were more evident for ZT10 dosing (Figure 9B). This may contribute to the superior anti-colitis effects of CR at ZT10 consistent with the expression of REV-ERBα (a higher expression at ZT10 than at ZT2) and the temporal berberine effects. In addition, we observed no significant difference in systemic exposure or colon distribution of berberine between ZT2 and ZT10 dosing of CR (Supplementary Figure S2). It was noted that PR treatment did not affect the expression of lipid-related genes (e.g., *Fasn, Acaca, Srebf1, Pparƴ, Pon1, Pparα*, *Cd36* and *Cpt2*) in the liver (Ahn et al., 2008) (Supplementary Figure S4B). Overall, the effect of CR on chronic colitis is dosing time-dependent in mice, which is potentially attributed to diurnal expression of the drug target REV-ERBα.

## Discussion

In this study, we have observed chronoeffect of PR on hyperhomocysteinemia in mice (Figure 3). PR dosed at ZT10 generated a stronger effect on hyperhomocysteinemia than drug dosed at ZT2 (Figure 3). *Rev-erbα* ablation in mice abolished the dosing time-dependency in PR effect (Figure 4). Moreover, PR increased the expression levels of the REV-ERBα target genes *Bhmt*, *Cbs* and *Cth* (three key genes involved in homocysteine catabolism), thereby down-regulating the homocysteine level and alleviating hyperhomocysteinemia in mice (Figure 5). We also found that CR attenuates chronic colitis in mice in a dosing time-dependent manner (Figure 7). ZT10 dosing generated a stronger anti-colitis effect as compared to ZT2 dosing (Figure 7). This is accompanied by lower production of colonic inflammatory cytokines (i.e., *Nlrp3*, *IL-1β*, *IL-6*, *Tnf-α* and *Ccl2*, all these are REV-ERBα target genes) in ZT10-treated than in ZT2-treated colitis mice (Figure 9). In addition, loss of *Rev-erbα* in mice abrogated the dosing time-dependency in CR effect (Figure 8). In our previous studies, puerarin (a main active constituent of PR) acts on the circadian protein REV-ERBα as an antagonist to alleviate hyperhomocysteinemia, whereas berberine (a main active constituent of CR) acts on REV-ERBα as an agonist to attenuate chronic colitis (Chen et al., 2020; Zhou et al., 2020). The diurnal patterns of PR and CR effects are respectively consistent with those of puerarin and berberine (Chen et al., 2020; Zhou et al., 2020). Therefore, we propose that the time-varying PR and CR effects may be attributed to the rhythmic expression of REV-ERBα as the drug target of the two herbal medicines.

The findings from current and our previous studies support the notion that targeting REV-ERBα represents a promising approach for prevention and management of colitis and hyperhomocysteinemia (Wang et al., 2018; Chen et al., 2020; Zhou et al., 2020). Besides these two types of diseases, REV-ERBα has been implicated in the development of many other disorders such as hypercholesterolemia, obesity, dyslipidemia, and diabetes (Zhang et al., 2018b; Yuan et al., 2019; Zhang et al., 2019). It was thus reasonable to postulate that PR and CR may also show pharmacological effects on these REV-ERBα-regulated diseases because they can alter REV-ERBα activities via their chemical constituents including puerarin and berberine. However, whether this is the case or note awaits further investigations.

Searching for the most appropriate timing for drug administration is the main goal of chronotherapeutics. Chronotherapy schedules with optimal dosing times have been developed and shown to be more effective than conventional treatment for various diseases such as cancers, cardiac and vascular diseases (Altinok et al., 2009; Smolensky et al., 2020). IBD is a chronic relapsing idiopathic disease characterized by epithelial barrier damage and inflammatory homeostasis disruption in the intestinal tract (Gupta et al., 2007; Wang et al., 2017). The pathogenesis of IBD remains poorly understood, and pharmacotherapy for the disease is far from optimal. In this regard, chronotherapy may provide a new means to improve treatment outcome against colitis. This is based on the facts that colitis displays a circadian rhythm in disease severity and that the effects of drugs (both berberine and CR) depend on the dosing time (Zhou et al., 2020; Figure 6). However, it is acknowledged that chronoeffects of CR and its active constituent berberine require validations in humans as current findings are based on experimental animals.

Anti-hyperhomocysteinemia effects of PR were assessed by measuring body tHcy and TG levels as previously reported (Chen et al., 2020). We used the doses of PR extract at 62.5, 125, and 188 mg/kg based on the extraction yield and dose translation between humans and mice (Nair et al., 2018). PR dose-dependently decreased the plasma and hepatic levels of both tHcy and TG in hyperhomocysteinemia mice (Figure 2). Although changes in tHcy and TG between 188 mg/kg PR and 125 mg/kg PR treatment are noted, the changes are not statistically significant (Figure 2). This suggests potential saturation in the pharmacological effects of PR. The exact reasons as to why this occurs are unknown. However, it is speculated that the saturation in solubility (i.e., the active ingredients may be not fully dissolved in the gastrointestinal tract) at the dose of 188 mg/kg may play a contributing role.

In the present study, CR doses (12.5, 25, and 50 mg/kg) and treatment duration (7 days) for pharmacological assessment against chronic colitis in mice were formulated according to a previous report (Jeong et al., 2014). The effect of CR on chronic colitis was accessed based on DAI, colon length, MDA/MPO activities and IL-1β/IL-6 levels as well as histopathological analysis. The parameters for DAI scoring here are comprehensive functional measures that are somewhat analogous to clinical symptoms observed in human IBD, and the scoring method has been validated by prior studies (Murthy et al., 1997). Colon length is significantly shortened in colitis mice, and represents a marker of intestinal inflammation. MPO is an enzyme existing in neutrophil leucocytes and has been found to be a reliable marker for the neutrophil accumulation in inflamed tissues (Wang et al., 2008). MDA is a product derived from the lipoperoxidative processes that take place as a consequence of the colonic oxidative insult (Wang et al., 2008; Zhou et al., 2020). The MDA level is much higher in colitis and often times used as an index of oxidative status in colitis (Zhou et al., 2020).

The chronoeffects of puerarin and berberine have been reported in our recent studies (Chen et al., 2020; Zhou et al., 2020). However, the drug effect and behaviors of single compound cannot be directly translated to the botanical drugs due to potential interferences from other ingredients (there may be major differences between herbal extract and pure single constituent in terms of pharmacokinetics and pharmacology). There is a need to further test whether the time-varying effects are applicable to the botanical drugs. In addition, chronoeffect of puerarin was reported previously only after intraperitoneal injection (Chen et al., 2020). Therefore, current chronoeffect study with *Puerariae radix* and *Coptidis rhizome* (oral administration) is more practical and meaningful because 1) herbal medicine as a whole rather than single constituent is used as a drug for disease treatment; and 2) botanical drugs are generally administered via the oral route.

Our previous studies have revealed that the level of Hcy and the severity of colitis (reflected by inflammatory factors) varied according to the time of day (Wang et al., 2018; Zhang et al., 2019). Circadian variation in the severity of disease is a potential factor contributing to time-varying drug effects. It was thus necessary to minimize this confounding factor as PR or CR effect on hyperhomocysteinemia or colitis were mainly measured by the changes in Hcy or inflammatory factors. To this end, we sampled PR- and CR-treated mice at the same time point (i.e., ZT2) after time-dependent treatments. By doing this, effects of disease related parameters (time-varying Hcy and inflammatory factors) on chronoeffects of PR and CR can be excluded. Such practice has been noted in chronoefficacy evaluation of aspirin (Tobias et al., 2015).

In conclusion, our study reveals the dosing time as a potential factor affecting the pharmacological effects of PR and CR. Further studies are suggested to investigate the use of PR and CR as chronotherapeutic treatments for humans. One limitation of current study is that overall impact of timed treatment on drug effects is unaddressed as only two dosing times are assessed.

## Data Availability

The raw data supporting the conclusion of this article will be made available by the authors, without undue reservation, to any qualified researcher.
