# Supplementary material for: Chronoeffects of the Herbal Medicines Puerariae radix and Coptidis rhizoma in Mice: A Potential Role of REV-ERBα
Source: Front Pharmacol. 2021 Jul 28;12:707844. doi: 10.3389/fphar.2021.707844 (PMC8355589; doi:10.3389/fphar.2021.707844)
Supplement: Supplementary file 1 [file DataSheet1.doc]

**Supplementary material**

# Materials and Methods

## Pharmacokinetic experiments

PR (125 mg/kg) was administered to wild-type mice (male, 6–8 weeks) by oral gavage at ZT2 or ZT10. At predetermined time points (5, 15, 30 and 60 min), mice (*n* = 3 per time point) were rendered unconscious with isoflurane. Blood samples were collected and centrifuged at 8000 g for 8 min to obtain plasma samples. Livers were immediately collected at 15 and 60 min. CR (50 mg/kg) was administered to wild-type mice (male, 6–8 weeks) by oral gavage at ZT2 or ZT10. At predetermined time points (30, 60, 90 and 120 min), mice (*n* = 3 per time point) were rendered unconscious with isoflurane. Blood samples were collected and centrifuged at 8000 g for 8 min to obtain plasma samples. Colons were immediately collected at 60 and 120 min. The tissue samples were homogenized using IKA T25-Digital Ultra-Turrax (Staufen, Germany). Plasma and tissue homogenates were mixed with acetonitrile (1:5, v/v), vortexed for 3 min, and centrifuged at 13,000 g for 15 min. The supernatant was dried using Eppendorf Concentrator Plus (Hamburg, Germany). The dry residue was reconstituted in 200 μl 50 % methanol (v/v), followed by vortex and centrifugation (13,000 g, 15 min). The supernatant was injected into a UPLC-QTOF/MS system for drug quantification (Waters, Milford, MA).

**Figure S1 Pharmacokinetic curves (A) and liver distribution (B) of puerarin in mice with hyperhomocysteinemia after PR (125 mg/kg, oral gavage) dosing at ZT2 or ZT10.**

**B**

**A**

**Figure S2 Pharmacokinetic curves (A) and colon distribution (B) of berberine in mice with chronic colitis after CR dosing (50 mg/kg, oral gavage) at ZT2 or ZT10. Data are mean ± SD (*n* = 3).**

**A**

**B**

**Figure S3 (A) Body weight curves of control and colitis mice during CR treatment. (B) Daily food intake of control, vehicle-treated, and CR-treated colitis mice. (C) Daily water consumption of control, vehicle-treated, and CR-treated colitis mice. Data are mean ± SD (*n* = 12).**

**
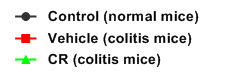
**

**A**

**B**

**C**

**Figure S4 (A) Effects of PR (125 mg/kg) on expression of the inflammatory factors *Nlrp3, IL-1β, IL-6, Tnf-α* and *Ccl2* in the colon of mice. (B) Effects of CR (50 mg/kg) on expression of the lipid-related genes *Fasn, Acaca, Srebf1, Pparƴ, Pon1, Pparα*, *Cd36* and *Cpt2* in the liver of mice. Data are mean ± SD (*n* = 5). Rel, Relative.**

**A**

**B**

**
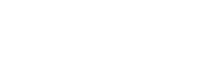
**
